# Supplementary material for: A Cocktail of Lipid Nanoparticle-mRNA Vaccines Broaden Immune Responses against β-Coronaviruses in a Murine Model
Source: Viruses. 2024 Mar 21;16(3):484. doi: 10.3390/v16030484 (PMC10976147; doi:10.3390/v16030484)
Supplement: Supplementary file 1 [file viruses-16-00484-s001.zip › viruses-2907849-supplementary.pdf]

Appendix A

Table S1. Mutation sites of the encoding mRNA from BA.4&5 and Delta

WHO label	Pango lineage	Mutation site
Delta	B.1.617.2	T19R, G142D*, 156del, 157del, R158G, L452R, T478K, D614G, P681R, D950N
Omicron	BA.4&5	A67V, D69–70, T95I, D143–145, G339D, S371L, S373P, K417N, N440K, G446S, S477N, T478K, E484A, G496S, Q498R, N501Y, P681H, T547K, D614G, H655Y, N679K, N764K, D796Y, N856K, Q954H, N969K, L452R, F486V

Table S2. Immunization schedule for sequential and cocktail immunization strategy

Immunization strategy	Group	Prime 100 µL	Boost 1 100 µL	Boost 2 100 µL
Sequential	S-M-O-D	S<br>6 µg	M<br>6 µg	O, D<br>6 µg/LNP-mRNA
Cocktail	OD	O, D<br>6 µg/LNP-mRNA	O, D<br>6 µg/LNP-mRNA	O, D<br>6 µg/LNP-mRNA
	SMOD(Hd)	S, M, O, D<br>6 µg/LNP-mRNA	S, M, O, D<br>6 µg/LNP-mRNA	S, M, O, D<br>6 µg/LNP-mRNA
	SMOD(Ld)	S, M, O, D<br>1.5 µg/LNP-mRNA	S, M, O, D<br>1.5 µg/LNP-mRNA	S, M, O, D<br>1.5 µg/LNP-mRNA
Control	Blank	PBS	PBS	PBS

1. Cao, Y.; Yisimayi, A.; Jian, F.; Song, W.; Xiao, T.; Wang, L.; Du, S.; Wang, J.; Li, Q.; Chen, X.; Yu, Y.; Wang, P.; Zhang, Z.; Liu, P.; An, R.; Hao, X.; Wang, Y.; Wang, J.; Feng, R.; Sun, H.; Zhao, L.; Zhang, W.; Zhao, D.; Zheng, J.; Yu, L.; Li, C.; Zhang, N.; Wang, R.; Niu, X.; Yang, S.; Song, X.; Chai, Y.; Hu, Y.; Shi, Y.; Zheng, L.; Li, Z.; Gu, Q.; Shao, F.; Huang, W.; Jin, R.; Shen, Z.; Wang, Y.; Wang, X.; Xiao, J.; Xie, X. S., BA.2.12.1, BA.4 and BA.5 escape antibodies elicited by Omicron infection. *Nature* **2022,** *608* (7923), 593-602.
